# Completeness of cancer registration: a new method for routine use

**DOI:** 10.1054/bjoc.1999.1048

**Published:** 2000-02-01

**Authors:** J Bullard, M P Coleman, D Robinson, J-M Lutz, J Bell, J Peto

**Affiliations:** Thames Cancer Registry, 1st Floor Capital House, Weston Street, London, SE1 3QD, UK; Section of Epidemiology, Institute of Cancer Research, Belmont, Surrey, SM2 5NG, UK

**Keywords:** cancer registration, completeness, death certificate only

## Abstract

We report a new method of estimating the completeness of cancer registration, in which the proportions of unregistered patients are derived from the time distributions of three probabilities, each of which can be directly estimated from the registry's own data – the probabilities of survival, of registration of the cancer during the patient's life, and of the mention of cancer on the death certificate of a cancer patient who dies. This method allows completeness to be assessed routinely by factors such as age, sex, geographical area and tumour type. © 2000 Cancer Research Campaign


British Journal of Cancer (2000) 82(5), 1111–1116
© 2000 Cancer Research Campaign
DOI: 10.1054/ bjoc.1999.1048, available online at http://www.idealibrary.com on IDEAL®

### Completeness of cancer registration: a new method for routine use

**J Bullard[1,*], MP Coleman[1,†], D Robinson[1], J-M Lutz[1,‡], J Bell[1] and J Peto[2]**

[1]Thames Cancer Registry, 1st Floor Capital House, Weston Street, London SE1 3QD, UK; [2]Section of Epidemiology, Institute of Cancer Research, Belmont, Surrey SM2 5NG, UK

**Summary** We report a new method of estimating the completeness of cancer registration, in which the proportions of unregistered patients are derived from the time distributions of three probabilities, each of which can be directly estimated from the registry's own data – the probabilities of survival, of registration of the cancer during the patient's life, and of the mention of cancer on the death certificate of a cancer patient who dies. This method allows completeness to be assessed routinely by factors such as age, sex, geographical area and tumour type.
© 2000 Cancer Research Campaign

**Keywords:** cancer registration; completeness; death certificate only

Population-based cancer registries form a valuable resource for public health and research, by providing information on the surveillance of cancer incidence and survival. The utility of such registries depends heavily on the completeness with which patients eligible for registration are ascertained, but registries rarely report their completeness because it is difficult to measure. Current methods of estimating completeness have several defects.

The most widely used approach employs indirect indices of the completeness of registration, such as the ratio of cancer incidence to mortality in a given period, or the percentage of 'death certificate only' (DCO) registrations (Parkin et al, 1994). The values of such indices can be compared with those obtained in other registries. However, they give only a broad indicator of quality, not an accurate estimate of completeness, and interpretation depends on assumptions about survival rates and the level of completeness in the other registries. Although the ratio of DCO to total registrations is widely used as an 'important check on the completeness of registration' (Parkin and Muir, 1993), Brenner (1995*a*) has shown that it is unreliable even as an indirect guide.

A second approach involves re-ascertainment of cases. The registry's staff search the medical records of all patients in a suitable sample of hospitals or other data sources for all registrable cancers. The proportion of eligible patients who are already registered is then a direct and quantitative estimate of completeness. However, this approach is too expensive and time-consuming for routine use, and only assesses the completeness of patients attending the data sources sampled. Results have rarely been published (Heiberger et al, 1983; Mattsson et al, 1985; Galceran et al, 1995).

A third approach involves obtaining an independent sample of cancer patients resident in the registry's territory, e.g. from cohort studies that do not rely on the same flagging system as the registry (Hunt and Coleman, 1987; Storm, 1988; Villard-Mackintosh et al, 1988; Hawkins and Swerdlow, 1992), or a series of clinical cases (Haddow, 1968; Larsson, 1971; Freedman, 1978; Nwene and Smith, 1982; Draper et al, 1989 Swerdlow et al, 1993; Warnakulasuriya et al, 1994). The proportion of eligible patients in this external sample who were also registered by the cancer registry provides an estimate of completeness. Some authors have used capture–recapture analyses to estimate completeness from two or more sources (Robles et al, 1988; Brenner, 1995*b*). This opportunistic approach is convenient and labour-saving, but relies heavily on the independence of the two data sources and this may be difficult to check. Moreover, such methods are unable to estimate the number of cases not routinely notified to the registry by any of the sources, and lack the statistical power to detect incompleteness at an early stage (Schouten et al, 1994).

The method proposed here addresses the defects of existing methods in several ways. It is based on the logical flow of data in the registration system, and on the time distribution of various probabilities inherent in this flow. It is thus in principle adaptable to cancer registries with different patterns of registration. It does not require re-abstraction of data, and can be executed rapidly and inexpensively, thus providing routine surveillance of completeness by variables such as tumour site, age, sex and geographic area.

### METHODS

The aim of cancer registries is to register cancers at, or soon after, diagnosis. However, some cases are missed. UK cancer registries obtain patient information from hospitals, pathology laboratories, GP practices, general cancer registries in other regions where members of their populations may have been treated and from specialist cancer registries restricted to a particular age group or tumour site. Additionally, copies of all death certificates mentioning malignant disease are sent by the Office for National Statistics (ONS) to the cancer registry.

*Received 28 April 1999*
*Revised 3 September 1999*
*Accepted 6 September 1999*

*Correspondence to:* J Bell

*Present address: Quintiles (UK), Ringside, 79 High Street, Bracknell, Berks RG12 1DZ, UK

†Present address: London School of Hygiene and Tropical Medicine, Keppel Street, London WC1E 7HT, UK

‡Present address: Registre genevois des tumeurs, 55 blvd de la Cluse, CH-1205 Geneva, Switzerland.

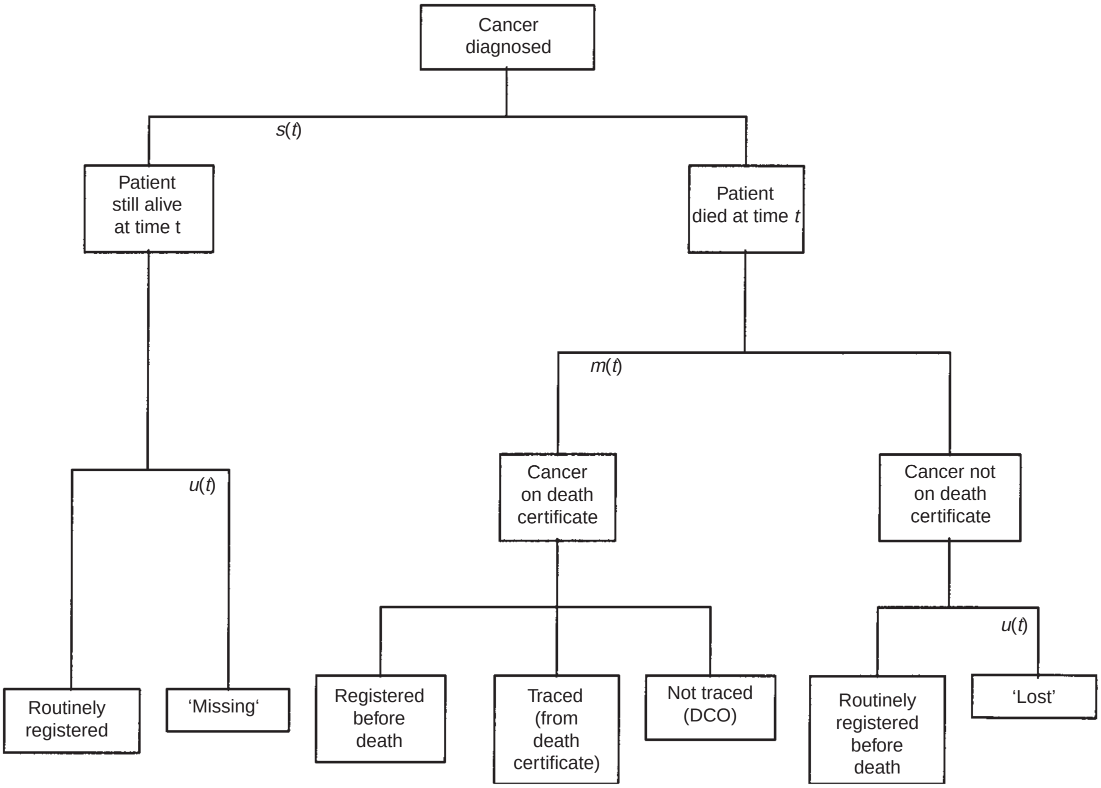

**Figure 1** Relationships between the various possible categories of registered and unregistered cancer patients at time *t* after diagnosis

The following methodology relies on the assumptions that death certificates are received for *all* patients dying with malignant disease mentioned on the death certificate, and that patients are not registered from sources other than death certificates after death. A search of the Thames Cancer Registry's database found no patients who were known from independent sources to have died for whom a death certificate had not been received from ONS. Moreover, only a fraction of a per cent of patients dying without cancer being mentioned on the death certificate (who would not have been registered initially from the death certificate) were registered after death.

Since cancer patients who subsequently die with cancer mentioned on the death certificate are (in the UK) routinely notified to the relevant registry, fatal cases are highly likely to be registered. The patients who remain unregistered at a given time after diagnosis of cancer are of two types. Firstly, patients who are alive and still unregistered (i.e. 'missing' from the register). Or patients who have died without being registered during life, and remain unregistered because the death certificate did not mention cancer. There is little chance of such patients ever being registered, and such cases may be described as 'lost' to the system. This process is illustrated in Figure 1.

We define three time-dependent probabilities, as follows:

$s(t_i)$ is the probability that a cancer patient is still surviving at time $t_i$ after diagnosis

$m(t_i)$ is the probability that the death certificate of a patient who dies in the time interval $(t_i, t_{i+1})$ after diagnosis includes a mention of cancer

$u(t_i)$ is the probability that a patient surviving until time $t_i$ after diagnosis is still unregistered.

We can then derive the proportions of 'missing' (M) and 'lost' (L) patients as follows:

1. Missing Proportion 'missing' at time $t_i$ after diagnosis is given by

$$M(t_i) = prob\ (surviving\ \underline{and}\ still\ being\ unregistered\ at\ time\ t_i) = s(t_i) \cdot u(t_i)$$

2. Lost From the survival distribution, the probability that a death occurs during the time interval $(t_i, t_{i+1})$ is

$$s(t_i) - s(t_{i+1})$$

Then *prob (death occurs in interval* $(t_i, t_{i+1})$ *and cancer not mentioned)*

$$= [s(t_i) - s(t_{i+1})] \cdot [1 - m(t_i)]$$

Since any patient who has died without being registered had clearly not been registered whilst still alive, it follows that

*prob (death occurs in interval* $(t_i, t_{i+1})$ *and patient not previously registered and cancer not mentioned on death certificate)*

$$= [s(t_i) - s(t_{i+1})] \cdot [1 - m(t_i)] \cdot u(t_i)$$

*© 2000 Cancer Research Campaign*

At time T after diagnosis, a cancer patient who is dead yet remains unregistered could have died at any time $t_i$ $(0<t_i<T)$. Thus the proportion 'lost' at time T is given by

$$L(T) = \text{prob (dead by time T \underline{and} unregistered \underline{and} cancer not mentioned)}$$
$$= \Sigma^{n}_{i=0} \{[s(t_i) - s(t_{i+1})] \cdot [1 - m(t_i)] \cdot u(t_i)\}$$

with $t_n \leq T < t_{n+1}$ and where $t_o$ $(=0)$ is the time of diagnosis.

Completeness at time T after diagnosis, $C(T)$, can now be found by subtracting from unity the proportions of patients who are 'missing' or 'lost', to give

$$C(T) = 1 - M(T) - L(T)$$
$$= 1 - s(t_n) \cdot u(t_n) - \Sigma^{n}_{i=0}\{[s(t_i) - s(t_{i+1})] \cdot [1 - m(t_i)] \cdot u(t_i)\}$$

also with $t_n \leq T < t_{n+1}$.

Thus, if reasonable estimates of the three time-dependent probabilities implicit in Figure 1 can be obtained, then completeness can be readily estimated. *s(t)* can be obtained from the distribution of survival times for registered patients, *m(t)* can be estimated from the death certificates of a sample of registered patients who have died, and *u(t)* from the distribution of the interval between diagnosis and registration.

### Probability of survival, *s(t)*

The survival distribution can be estimated using the actuarial method (Cutler and Ederer, 1958; Esteve et al, 1994). Convention amongst cancer registries is to calculate survival for all cancers diagnosed in a given period among residents of its territory. Patients registered solely from a death certificate (DCO cases) are usually excluded because their date of diagnosis is unknown. However, this practice can lead to bias. Berrino et al (1995) have shown that patients dying with cancer mentioned on the death certificate have poorer survival than patients dying of other causes, and that the percentage reduction in estimated survival resulting from the inclusion of DCO cases is generally of the same order as the proportion of such cases in the series under study. If possible, DCOs should be included as outlined below.

It is important to estimate the survival distribution for patients rather than tumours. Some 5–10% of cancer patients will eventually develop more than one primary, but the criteria for registering a second primary vary between cancer registries (Parkin et al, 1994). It is in any case intuitively sensible to include each patient only once, using the date of registration of the first tumour.

It is not clear how DCOs should be included in estimates of survival, since both their date of diagnosis and their duration of survival are unknown. To address this problem a working assumption was made that, for any given period, the unknown number of cancer patients whose true date of diagnosis lies within that period, but who will eventually be registered as DCOs, is the same as the number of DCOs recorded as dying in that period. If the survival distribution of cases diagnosed in 1987 were required, for example, the estimate would be based on non-DCO cases *diagnosed* in 1987 and DCO cases *dying* in 1987.

DCOs are distinguished from other death-certificate-initiated (DCI) cases only in that the registry has not succeeded in tracing the patient's medical record to obtain the date of diagnosis. It therefore seems reasonable to apply the survival distribution of successfully traced-back DCI patients to those for whom trace-back was unsuccessful, i.e. the DCO cases.

The survival times for DCO cases can therefore be approximated by the survival distribution of those DCI patients whose details were successfully traced back from the death certificate *and who were diagnosed in the required period*, specific for age at death, sex, tumour type and geographic area.

It may be possible to refine the method further by categorizing dead cases by place of death (e.g. hospital, hospice, home, etc.). However, this idea has not been pursued in the current study.

### Probability that cancer is mentioned on the death certificate, *m(t)*

This can be estimated from cancer registrations for patients who are now dead. The same sample of patients used to estimate the survival distribution can also be used for this purpose. The numerator is the number of deaths in the given interval since diagnosis for which the death certificate includes a mention of cancer, while the denominator is the total number of deaths in the same interval. The simplest approach is to use patients diagnosed in a given calendar period who are now dead. This will tend to overestimate the true probability of cancer being mentioned on the death certificate, since all patients who die of their cancers will be registered, whilst some patients who die of other causes will not.

### Probability of failure of registration before death, *u(t)*

The cancer registry receives copies of all death certificates mentioning cancer for deaths in its territory, irrespective of whether the patient has already been registered. Crucially, this source of information depends on civil death registration, and is completely independent from the sources of routine cancer registration. These certificates enable *u(t)* to be calculated for cancer deaths occurring in a given period.

The periods from diagnosis to registration and from diagnosis to death are calculated (with estimates for DCOs being made as described above). The probability of a patient not being registered before death can then be estimated with the same approach as for survival, but treating registration before death as the 'event', and censoring at death.

This was refined by censoring at 1 year before death, because the probability of registration among those who die of cancer increases during the year or so before death. The death certificates of patients sampled to estimate *u(t)* contain a mention of cancer, whereas *u(t)* is to be applied to survivors and to patients whose death certificate does not mention cancer. Censoring 1 year before death removes most of this excess probability, and the resultant distribution is more suitable for non-cancer deaths.

### RESULTS

The survival distribution *s(t)* was based on 56 992 cases 'diagnosed' in 1987, including 14 409 DCO cases dying in 1987, but excluding non-melanoma skin cancers and patients resident outside the registry's territory. Five years after diagnosis, almost 80% of these patients had died (Figure 2).

*m(t)* was calculated from the records of patients diagnosed in 1987 who had died by the end of 1993. The data comprised 42 971 patients (including 14 409 DCOs) whose death certificate did mention cancer and 2620 patients whose death certificate did not. In the period immediately after diagnosis, as expected, most death certificates mentioned cancer, but this proportion fell to approximately 80% by 5 years after diagnosis.

© 2000 Cancer Research Campaign 

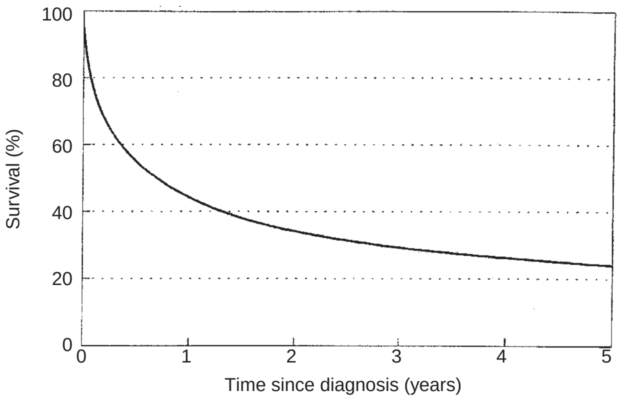

**Figure 2** The estimated survival distribution *s(t)* for all cancer patients (excluding non-melanoma skin cancer) diagnosed in 1987

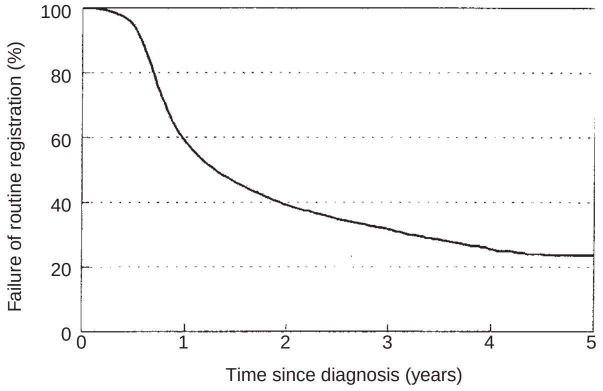

**Figure 3** The probability that a surviving cancer patient remains unregistered by time since diagnosis, *u(t)*

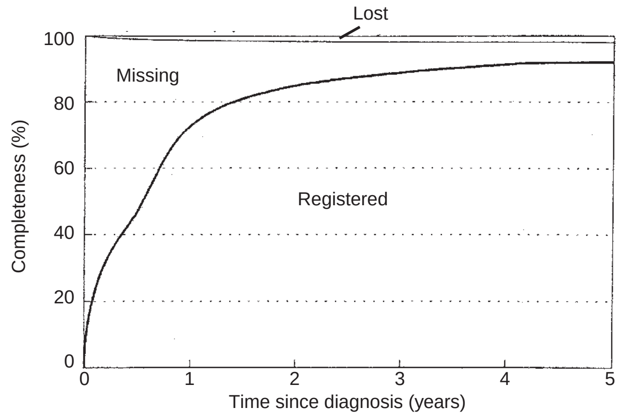

**Figure 4** The completeness of cancer registration *C(T)* at Thames Cancer Registry for all cancers diagnosed in 1987 (excluding non-melanoma skin cancer) by time since diagnosis

The probability of failure of registration before death, *u(t)*, was calculated from all cancer deaths recorded at the registry during 1991. As before, patients with non-melanoma skin cancer and patients resident outside the registry's territory were excluded. The 45 384 cancer deaths comprised 9744 (21.5%) registrations made before death, 28 327 (62.4%) patients whose registration was made from the medical records after receipt of a death certificate and 7313 (16.1%) DCOs. In the first 6 months after diagnosis,very few surviving cases were registered (Figure 3). Most registrations of survivors occurred between 6 months and 2 years after diagnosis, and the probability that a surviving case remained unregistered gradually fell to 23% at 5 years after diagnosis.

Since this study was performed, improvements to the computerized matching of records together with active tracing of unmatched deaths have reduced the DCO rate at Thames Cancer Registry to 10.5% at the end of 1998.

Figure 4 shows the derived distribution of completeness *C(T)* with time for Thames Cancer Registry. The area above the bold curve represents the patients who remain unregistered at any given time. The thinner line divides these unregistered patients into those who are still alive (the 'missing') and those who have died without cancer being mentioned on the death certificate, and who are unlikely ever to be registered (the 'lost'). The results indicate that Thames Cancer Registry attains 92.1% completeness 5 years after diagnosis for all cancers.

The same method was used to calculate completeness by age and cancer site. The samples used to calculate the distributions *s(t)*, *m(t)* and *u(t)* were restricted to the age group or cancer site of interest. Otherwise, the calculations were performed exactly as above.

For estimation of completeness by age at diagnosis, the selection of DCOs causes difficulty, because the age at diagnosis is unknown. However, since age at death is available, we can apply the survival distribution of those patients whose date of diagnosis and duration of survival are known as a result of trace-back from the death certificate to each DCO in the same category of age at death. (We used the conventional 5-year age groups.) The estimated age at diagnosis for each DCO can then be found by subtraction. Those patients registered as a DCO whose estimated age at diagnosis falls outside the specified range are then ignored. The rest of the calculations are as before, within each age group at diagnosis.

Figure 5A shows the pattern of completeness by time since diagnosis for selected cancer sites. Registration appears most complete for lung cancer, the most lethal of those shown. Melanoma of the skin is the least complete of the selected sites. Whilst breast cancer and melanoma have similar survival distributions *s(t)*, *u(t)* is higher for melanoma, resulting in lower completeness.

Figure 5B shows the results for different age groups. In general, completeness is higher at older ages due to the fact that survival rates are lower in older patients, and death certificates can be used to improve case ascertainment. The only exception appears to be that childhood cancers (0–14 years) are more completely registered than those in young adults. This is due to greater efficiency of the routine registration procedures for the youngest patients.

Completeness of registration for other combinations of factors can be obtained by a suitable choice of the samples for estimating the three probability distributions.

### DISCUSSION

One advantage of this method over others in common use is that it shows how the completeness of registration of cancer patients diagnosed in a given period increases with time since diagnosis. Another advantage is the inclusion of an estimate of the percentage of cancer patients who are likely never to be registered (the 'lost' group). Further, the interpretation of this estimate of

© 2000 Cancer Research Campaign

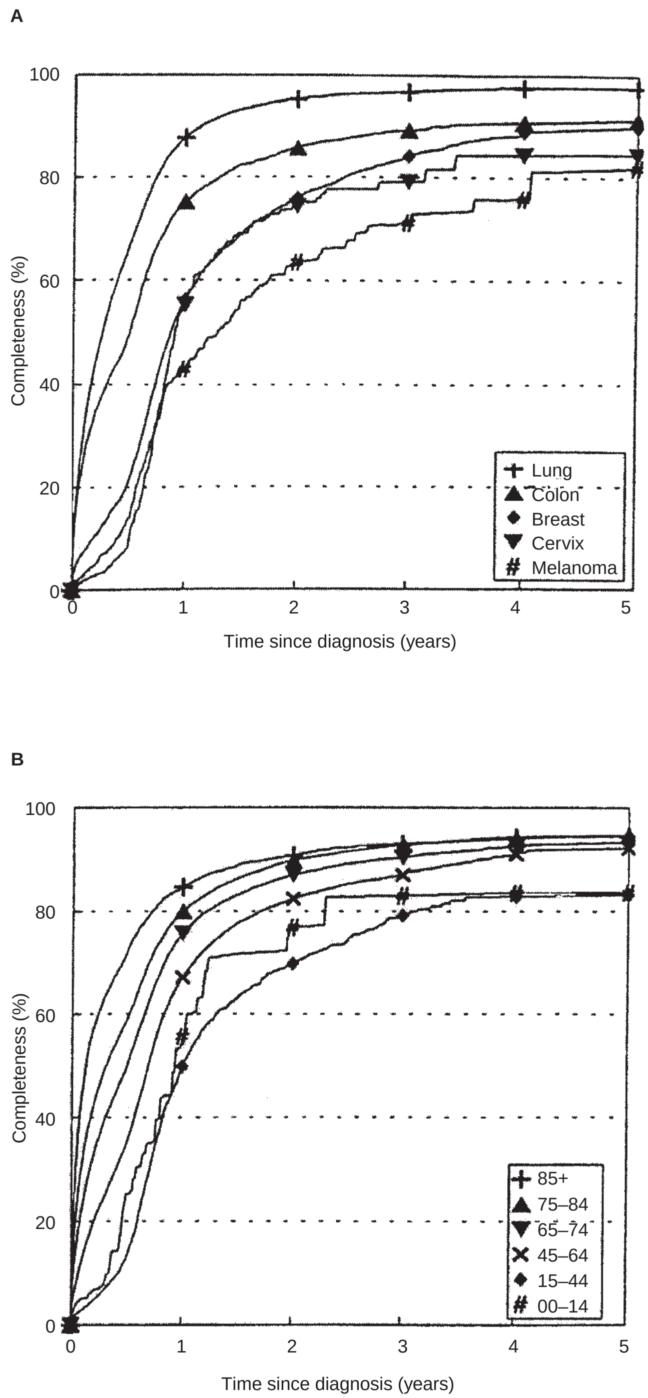

**Figure 5** The completeness of cancer registration by time since diagnosis: (**A**) by cancer site; (**B**) by age at diagnosis completeness does not depend on assumptions about the efficiency of other cancer registries (as with indirect indices) or other data sources (independent case ascertainment).

Three crucial requirements must be met for the method to be applicable. First, the date when each cancer is first registered must be systematically recorded. This date should not be changed subsequently, regardless of any further patient information that may be acquired. Although most of the UK cancer registries do record this date, it would be desirable to include it in any future national minimum data set. If dates of registration have only been recorded for the past *T* years, then *u(t)* and hence completeness can only be calculated up to *T* years after diagnosis. Ideally, the date of registration should be available for 3 or more years before the method is implemented, in order to ensure that the probability distribution of registration before death is estimated for a reasonable period.

Secondly, it is also essential that copies of all death certificates mentioning cancer should be received. This may be a problem for small regional registries in countries where there is no national follow-up system or matching with a national death index.

Thirdly, knowledge of whether each case is DCI is also required, in order to estimate the survival times for DCO cases as described above.

The method should improve comparability between cancer registries, since estimates of completeness could now refer to standard intervals such as 1, 2 or 5 years after diagnosis, and should enable more systematic exploration of variation in completeness by factors such as age, sex and type of tumour. However, the estimate of completeness should not be over-interpreted if the numbers used in the samples to calculate the underlying distributions are small. The effects of smaller numbers can be seen in the 'lumpiness' of some of the estimates presented in Figure 5.

Treatment of cancer patients is continually improving, leading to increased survival and an ever greater proportion of patients being 'cured' to eventually die of other causes, with no mention of cancer on the death certificate. Unless registration procedures improve, this will result in decreased levels of completeness. Incompleteness results in biased estimates of cancer incidence, prevalence and survival. The routine publication of completeness figures for standard intervals would aid the interpretation of such measures.

### ACKNOWLEDGEMENTS

This work was carried out at Thames Cancer Registry with funding from South West Thames Regional Health Authority's Locally Organised Research Fund. The authors would like to thank Stephen Darlington for help with computing and graphics, and Bendix Carstensen, Roger Black and Eric Holowaty for their helpful comments and suggestions.

### REFERENCES

Berrino F, Estève J and Coleman MP (1995) Basic issues in estimating and comparing the survival of cancer patients. In: *Survival of Cancer Patients in Europe: the EUROCARE Study*, Berrino F, Sant M, Verdecchia A, Capocaccia R, Hakulinen T and Estève J (eds), pp. 1–14. IARC Scientific Publication No. 132. IARC: Lyon

Brenner H (1995*a*) Limitations of the death certificate only index as a measure of incompleteness of cancer registration. *Br J Cancer* **72:** 506–510

Brenner H (1995*b*) Use and limitations of the capture–recapture method in disease monitoring with two dependent sources. *Epidemiology* **6**: 42–48

Cutler SJ and Ederer F (1958) Maximum utilisation of the life table method in analysing survival. *J Chron Dis* **8**: 699–712

Draper GJ, Bower BD, Darby SC and Doll R (1989) Completeness of registration of childhood leukaemia near nuclear installations and elsewhere in the Oxford region. *Br Med J* **299**: 952–952

Estève J, Benhamou E and Raymond L (1994) *Statistical Methods in Cancer Research, Volume IV. Descriptive Epidemiology*. IARC Scientific Publication No. 128. IARC: Lyon

Freedman LS (1978) Variations in the level of reporting by hospitals to a regional cancer registry. *Br J Cancer* **37**: 861–865

*© 2000 Cancer Research Campaign*

Galceran J, Porta M, Martin M, Mariné E and Borràs J (1995) *Completeness of Cancer Registration in Tarragona, 1980–1989: an Independent Case Ascertainment Study*, pp. 99. International Association of Cancer Registries: Rio de Janeiro

Haddow AJ (1968) The coverage achieved by the cancer registration scheme in the Western Hospital Region of Scotland in 1965. *Health Bull (Edinburgh)* **26**: 10–12

Hawkins MM and Swerdlow AJ (1992) Completeness of cancer and death follow-up obtained through the National Health Service Central Register for England and Wales. *Br J Cancer* **66**: 408–413

Heiberger RM, Miller CL, Feigl P, Lane WW and Glaefke G (1983) A novel method of assessing completeness of tumor registration. *Cancer* **51**: 2362–2366

Hunt K and Coleman MP (1987) The completeness of cancer registration in follow-up studies – a cautionary note. *Br J Cancer* **56**: 357–359

Larsson S (1971) Completeness and reliability of lung cancer registration in the Swedish Cancer Registry. *Acta Pathol Microbiol Immunol Scand* **79**: 389–398

Mattsson B, Rutqvist LE and Wallgren A (1985) Under-notification of diagnosed cancer cases to the Stockholm cancer registry. *Int J Epidemiol* **14**: 64–69

Nwene UP and Smith A (1982) Assessing completeness of cancer registration in the north-western region of England by a method of independent comparison. *Br J Cancer* **46**: 635–639

Parkin DM, Chen VW, Ferlay J, Galceran J, Storm HH and Whelan SL (1994) *Comparability and Quality Control in Cancer Registration*. IARC Technical Report No. 19. IARC: Lyon

Parkin DM and Muir CS (1993) Comparability and quality of data. In: *Cancer Incidence in Five Continents, Volume VI*, Parkin DM, Muir CS, Whelan SL, Gao Y-T, Ferlay J and Powell J (eds). IARC Scientific Publication No. 120. IARC: Lyon

Robles SC, Marrett LD, Clarke EA and Risch HA (1988) An application of capture–recapture methods to the estimation of completeness of cancer registration. *J Clin Epidemiol* **41**: 495–501

Schouten LJ, Straatman H, Kiemeney LALM, Gimbrère CHF and Verbeek ALM (1994) The capture–recapture method for estimation of cancer registry completeness: a useful tool? *Int J Epidemiol* **23**: 1111–1116

Storm HH (1988) Completeness of cancer registration in Denmark 1943–1966 and efficacy of record linkage procedures. *Int J Epidemiol* **17**: 44–49

Swerdlow AJ, Douglas AJ, Vaughan Hudson G and Vaughan Hudson B (1993) Completeness of cancer registration in England and Wales: an assessment based on 2145 patients with Hodgkin's disease independently registered by the British National Lymphoma Investigation. *Br J Cancer* **67**: 326–329

Villard-Mackintosh L, Coleman MP and Vessey MP (1988) The completeness of cancer registration in England: an assessment from the Oxford-FPA contraceptive study. *Br J Cancer* **58**: 507–511

Warnakulasuriya KAAS, Acworth P, Bell CMJ and Johnson NW (1994) Incompleteness of oral cancer registration in south-east England, 1971–87. *Br J Cancer* **70**: 736–738

*© 2000 Cancer Research Campaign*
